# Effects of Play in an Upright Position on Intra-Individual Variability of Gross Motor and Language Development in Institutionalized Infants

**DOI:** 10.3390/ijerph191811804

**Published:** 2022-09-19

**Authors:** Sunanta Prommin, Wantana Siritaratiwat, Surussawadi Bennett, Lugkana Mato, Orawan Keeratisiroj, Worawan Kamruecha

**Affiliations:** 1 Research Center in Back, Neck, Other Joint Pain and Human Performance (BNOJPH), Faculty of Associated Medical Sciences, Khon Kaen University, Muang, Khon Kaen 40002, Thailand; 2 School of Physical Therapy, Faculty of Associated Medical Sciences, Khon Kaen University, Muang, Khon Kaen 40002, Thailand; 3 Faculty of Public Health, Naresuan University, Phitsanulok 65000, Thailand

**Keywords:** play in upright position, gross motor development, language development, orphanage

## Abstract

(1) Objective: To investigate the effects of play in an upright position on intra-individual variability and to examine the relationship between the variability of gross motor and language development in institutionalized infants aged six to ten months. (2) Methods: Thirty infants were conveniently enrolled in either the experimental or control groups. The Alberta Infant Motor Scale (AIMS) and the Communication and Symbolic Behavior Scales Developmental Profile (CSBS-DP) Infant/Toddler Checklist were tested pre and post each monthly intervention for three months. Sixteen infants in the experimental group received an additional program of 45 min play in an upright position three times a week for a 3-month period. (3) Results: There were significant between-group differences in intra-individual variability of the AIMS percentiles (*p*-value = 0.042). In addition, there was a significant difference in the intra-individual variability of the language percentile between groups (*p*-value = 0.009). The intra-individual variability of gross motor development was significantly correlated (r_s_ = 0.541; *p* = 0.03) with language development. (4) Conclusions: Play in an upright position could be applied to improve intra-individual variability in gross motor and language development percentiles in institutionalized infants.

## 1. Introduction

A rising number of infants being brought into the orphanage with inadequate caregivers and resources results in the demanding duty of taking care of orphaned infants [1]. Most children’s homes or residential care facilities around the world are facing hardship due to an inappropriate ratio of caregivers to children, rotational shift work, or insufficient resources [2]. Infants in the orphanage thus have little caregiver–child interaction or less opportunity to experience movement [3]. Restricted opportunities to experience movement and environmental interaction within the context of orphanages may be linked with developmental motor and language delays.

There is reported evidence from cross-sectional research that institutionalized infants have poorer gross motor [4,5] and language [5] development than family-reared children. Moreover, 50% of residential children in orphanages have delayed motor development during their first three years of life [6]. These infants in orphanages usually experience social-emotional distress [7,8] and display delayed motor skills [9]. Limited caregiver–child interactions and environmental factors give rise to global developmental regression and cause a long-term relationship between development and mental deficits.

The emergence of skills in the developmental domain is assumed to be varied and involves the practice of functional ability in the active movement of infants [10]. The study of Darrah et al. (1998) showed that healthy infants naturally exhibit a large rate of change in gross motor development from birth up to 13 months [11]. Moreover, Darrah et al. (2003 and 2009) found that there is an instability regarding the motor and communication development percentile of typically developing infants from age 9 to 21 months [12,13]. Development is a dynamic process, and variability in development is thus not only dependent on the maturation of the CNS but also the result of the interaction of multiple subsystems within the child, the environment, the demands of the task, and the experience or practice of movement [14].

According to the commentary theory of motor development [14], the general concept and term used to describe intra-individual variability is an instability of the developmental percentile or dynamic changes within individuals over time. This intra-individual variability of development can be seen as the random swinging up or down of the developmental percentile [11] or changes in percentiles that can be calculated as the maximum minus the minimal value of the developmental percentile over a period of assessment time [15]. Cohen et al., in 2008, studied the motor and language longitudinal developmental delays of adopted infants at 6 weeks and 6 months follow-up. However, this study did not investigate intra-individual variabilities of motor and language development [16]. Previous studies have suggested that instability or intra-individual variability of developmental percentiles exhibited through longitudinal or series assessments and delayed development should not be determined through cross-sectional assessment [11,12].

The environment of an orphanage is rather restricted, as there is often crowding of infants in a limited space, the infants are raised in closed buildings, and the infants spend most of their time in their cots. Tirella et al. confirmed the lack of interaction in child residential facilities in Russia, where infants spent up to 65% of their time without any human interaction [17]. Although orphans sometimes have the opportunity to move independently or have definite activities in their daily living schedules, these infants have no or little opportunity for free play or exploring the outside environment surrounding them. Moreover, caregiver–child interactions tend to be limited due to overload, especially during the first year of life. Infants have rare opportunities to actively play outdoors compared with home-raised infants [1]. A previous prospective cohort study of 49 infants in Southern Brazil found that variability in motor and cognitive development was strongly associated with environmental risk factors. The environment in which child-rearing occurs hence has great potential to influence infant development and should be the essential target of interventional study programs [18].

Environments that are deprived may also impede motivation to move, which influences the emergence of new gross motor skills in infants. At the present, the study of intra-individual variability via longitudinal assessments of gross motor and language development of infants living in deprived environments is limited. As mentioned, a previous prospective cohort study in 49 infants from Southern Brazil found that variability in motor and cognitive development was strongly associated with environmental risk factors [18]. Our previous studies of gross motor development of infants residing in an orphanage environment found that institutionalized infants displayed less intra-individual variability in gross motor development [19,20]. These studies suggest that the environment of child-rearing has great potential to influence infant development and should be an essential target of intervention studies programs [18]. Therefore, intervention that modifies the institutional environment by increasing the quality of caregiver–child interaction, medical care, sanitation, and safety has led to substantial improvements in child development [3].

To promote child development of institutionalized infants, previous research has investigated the effects of caregiver training and environmental adaptation. Koch and Franzsen, in 2017, found that caregiver training significantly changed the amount of time that infants spent alone and time spent with their caregiver [21]. A systematic review by Hermanau et al. in 2016 reported the effects of structural interventions and caregiver training on child development in institutional environments [22]. They found that most studies examined the beneficial effects on the children’s emotional, social, and cognitive development, whereas few studies focused on the effects of interventions on the child–caregiver relationship or the general institutional environment. Furthermore, the effects of stimulation or the play program on developmental outcomes for infants in orphanages have been conducted [23,24,25,26,27]. Siritaratiwat et al. (2011) found that a massage program can be beneficial for healthy institutionalized infants aged less than 5 months. In order to promote a good range of gross motor development, evidence from research suggests that healthy infants should receive opportunities to actively participate in upright positions and explore the environment outside buildings [23]. In 2002, Taneja et al. developed a structured 90 min play program for orphaned infants and children aged 6 months to 2.5 years. This study showed that short daily sessions of play could significantly improve the motor, language, and cognitive development of children living in institutions [24]. Later, in 2004, Taneja et al. validated the 90 min program of daily structured play for infants in different orphanages [25]. The mean motor, language, and cognitive scores of children in orphanages rose dramatically after initiating a program of play. The program was validated in healthy children for other orphanages for 1 year in India. The results showed that this project could sustain motor, language, and cognitive development scores over long periods [26]. However, studies were conducted using a quasi-experimental design. Additionally, Berument et al. (2011) developed a program for improving the language and cognitive development of infants and young children residing in institutional settings in Turkey. The results indicated that a supporting program for child and caregiver interaction, such as singing children’s songs, reading children’s books, finger games, and playing with toys, could increase the quality of care of infants in children’s homes [27].

Apart from gross motor development, language development is a common concern for children in orphanages. Previous research studies have predicted language skills based on early gross motor development [28,29]. One study found that gross motor development is related to the appearance of later communication milestones [30]. LeBarton et al. suggested that the emergence of motor skills at age 3–5 months is a significant predictor of subsequent language development at age 10–14 months. Furthermore, LeBarton and Iverson, in 2016, found that at-risk infants aged 5–10 months who had a sibling with autism spectrum disorder tended to show delayed gross motor development related to communication delay. It could be assumed that orphaned infants living in restricted environments with poor gross motor development would show slow language development [30]. However, Darrah et al. found no relationship between gross motor and language skills in terms of the intra-variability of developments. They suggested that each domain of development is independent, and, therefore, early development in one domain cannot predict the development of the other domain at a later age [12]. Series assessments from previous studies found that gross motor development displays variability within individuals during the first 13 months of age, while language has intra-variability over 13 months onwards [11,12,13]. Based on this controversy, the infants with risk of early motor and communication delays should be longitudinally observed via serial assessments, and the investigation of the correlation of intra-individual variability between gross motor skill and language development in orphaned infants at the same age is needed.

To the best of our knowledge, there is currently still limited research investigating the effect of interventions on infant development at orphanages, and there are no experimental studies reporting on the effects of participating in a program of active play in an upright position and the correlation between the intra-variability of gross motor skills and language developments in infants living in orphanages. Therefore, this study aimed to investigate the effect of play in an upright position and the correlation between the intra-individual variability of gross motor skills and language development in institutionalized infants. The intra-individual variability of developmental percentiles in the current study was collected as the changes in the developmental percentile within individuals from the age of 6 to 10 months. We hypothesized that (1) the upright play program could alter intra-individual variability of gross motor and language percentiles of orphaned infants from age 6 to 10 months and (2) intra-individual variability between gross motor and language percentiles would be correlated in orphaned infants aged 6 to 10 months.

## 2. Materials and Methods

### 2.1. Study Site and Population

The population was infants residing in orphanages. The children’s home in Khon Kaen raises children from birth to 6 years. The children’s home at the Nakhon Si Thammarat and Songkla Province takes care of children from newborn to 18 years, especially abandoned infants who are admitted since birth or when found. Each orphanage has a homogenous system consisting of children of both genders, the cause of admission, and the origin of the child. Newborn to 12-month-old infants in all orphanages are taken care of in a similar physical environment, such as being raised in a closed setting of a two-story building or having little or no chance to be outside. Infant caring is divided into two working shifts per day or 12 h per work period. The caregiver-to-infant ratio is approximately 1 to 7–12. Infants aged more than 12 months are moved to a new room for ages 1 to 3 years and taken care of by different caregivers. In addition, the experienced caregivers in both groups used to receive training in baby massage and child-rearing practices from the Montessori foundation. However, both groups have a heterogeneous system based on visual observation that the main caregiver in the experimental group seemed to display attitudes and behaviors toward a child, such as singing a song and eye gaze, during daily activity more so than in the interaction between caregivers and infants in the control group.

### 2.2. Study Design and Participants

The current study was conducted according to a purposive cluster trial design with blinded assessors and contained two groups of infants being raised in orphanages. Infants who met the inclusion criteria were recruited from the Khaenthong children’s home in the northeastern part of Thailand for the experimental group and from the Srithammarat and the Songkla children’s home in the southern part of Thailand for the control group. The required sample size was calculated from the pilot study using the mean and standard of deviation of intra-individual variability of the gross motor and language percentile in 10 orphaned infants (5 infants per group) using the computer program G*power version 3.1.9.7 program (Institut für Experimentelle Psychologie, University in Düsseldorf, Düsseldorf, Germany) [31]. The power of the test was set at 0.80 for the significance level below 0.05 (Z*_α_*_/2_ = 1.96). The study also assumed 10% participant drop out [n_adj_ = 13/(1 − 0.1)^2^] [32] [n = 13 and R = 0.1]. Therefore, 32 orphaned infants were required for this study, with 16 infants in each group.

The inclusion criteria were (1) gestation age from 37 to 42 weeks, (2) birth weight ≥ 2.500 g, (3) Apgar score at 5 min between 8 and 10 [33], (4) aged 6 or 7 months at the time of recruitment, and (5) no history of receiving breast milk feeding. The exclusion criteria were (1) major neurological abnormalities such as muscle paralysis, abnormal muscle tone, or seizures, (2) genetic diseases such as Down’s syndrome or congenital diseases such as congenital heart disease and musculoskeletal disorders or physical deformities, and (3) visual and hearing deficits with other problems affecting global development. The withdrawal criteria were (1) being taken home by the natural parents or being adopted and (2) having other reasons inhibiting them from playing in an upright position for more than 7 of the 36 times for the experimental group [34].

**Figure 1:**
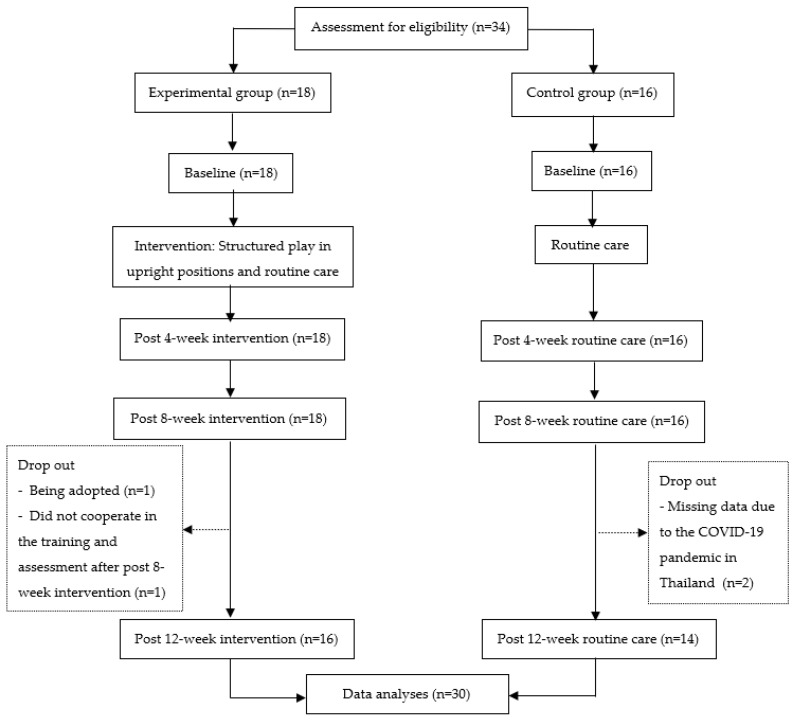
Flowchart of participant enrollment and analysis.

The assessment diagram for the eligibility of participants is shown in Figure 1. Thirty-four infants were recruited; however, 4 participants were excluded from the data analyses due to missing data in relation to the COVID-19 pandemic in March 2020. Data collection could not be performed on 2 infants from the control group. One infant from the experimental group was adopted by a family at the age of 8 months, and the other infant was able to cooperate in an upright position only 24 times. Data of the remaining 30 participants were then analyzed, including 16 participants in the experimental group and 14 infants in the control group.

The birth characteristics of participants from both groups were similar (Table 1). From birth characteristics, all participants were healthy full-term infants with no biological deficits. Infants in the experimental group contained more boys than girls, and these infants were admitted to the orphanage at an earlier age compared with those in the control group. Infants in orphanages were taken care of by caregivers of a similar age, level of education, and years of working experience (Table 2). There were high caregiver-to-infant ratios of 1 to 7–12 for each working shift.

### 2.3. Ethical Clearance

The study protocol was approved by the Ethics Committee in Human Research, Khon Kaen University, based on the Declaration of Helsinki and the ICH Good Clinical Practice Guideline (Institutional Review Board Number: IRB00008614, Protocol ID No: HE622234, 4 December 2019). The guardians of the orphanages signed informed consent allowing data collection at the orphanages and voluntary participation of their staff and infants to be observed the gross motor development prior to data collection. The trial was registered on Clinicaltrials.gov (number: TCTR20190930005). Throughout the study period, the heads of orphanages or staff could withdraw their infants from the study at any time they desired. The data were anonymized in order to not reveal participants’ identities, and the analyses were conducted in a way that the final results could not be linked to individuals.

### 2.4. Measurements

#### 2.4.1. Alberta Infant Motor Scale (AIMS)

The AIMS is a standard observational screening tool used to examine the gross motor movement in infants during the first 18 months or until infants can walk independently. The AIMS consists of 4 main positions with 58 items: 21 prone items, 9 supine items, 12 sitting items, and 16 standing items. This observational assessment requires minimal infant touching, and toys can be used to motivate movement. In the scoring of gross motor movement, 1 point is assigned to observed and 0 points to non-observed items displayed within the window of movement. Items below a window are counted as the previous items and credited with 1 point for each item. The scoring for each main position is summed from the items observed within the window of movement and the previous items credited [35]. Finally, the total raw scores can be converted to gross motor percentiles obtained from the previous study of 574 Thai full-term samples [36].

The observation of gross motor development in the current study was performed by a physiotherapist with 6 years of experience in pediatric physiotherapy using the Thai version of AIMS [37]. The assessor practiced using AIMS for 6 months with an expert who is familiar with the AIMS and has more than 10 years of clinical experience in delayed and disabled children. The inter-rater reliability between the assessor and the expert was assessed using video recordings of 10 full-term infants aged 6–12 months. Inter-rater reliability was analyzed using the intraclass correlation coefficient (ICC) model (3,1), showing an ICC value of 0.98 (95% CI = 0.95–0.99). The intra-rater reliability of the tester was determined prior to data collection and based on a one-month interval, and analysis using the ICC (3,1) showed an ICC value of 0.99 (95% CI = 0.98–0.99).

#### 2.4.2. Communication and Symbolic Behavior Scales Developmental Profile (CSBS-DP) Infant/Toddler Checklist

The Communication Symbolic Behavior Scales Developmental Profile (CSBS-DP) Infant/Toddler Checklist was used in the current study to evaluate the communication abilities of infants by the main caregiver who is familiar with the child in daily living. The CSBS-DP is a descriptive ordinal scale developed to identify the social, speech, and symbolic composite functions of infants aged 6 to 24 months. There are seven clusters scored for the summation of 57 scores. The scoring of each item ranges from 0 points for a ‘Not Yet’ item, 1 point for a ‘Sometimes’ item, to 2 points for an ‘Often’ item. Items describing a series of numbers are scored as 0 points for a ‘None’ item and 1–4 points for items containing numbered choices. The obtained total scores are changed to the standard norm-referenced percentiles. Normative data of the CSBS-DP checklist were generated from 2188 infants aged 6–24 months from a total of eight sites in the United States of America and two sites in Canada. Cronbach’s alpha coefficient is 0.93 for 167 children with normal development [38].

### 2.5. Interventions

#### 2.5.1. Structured Play in Upright Positions

Infants in the experimental group received an additional program of 45 min structured play, 3 days per week for 12 weeks. The 45 min structured program contained a 30 min play session in an upright posture and a 15 min free movement session on the floor. The 30 min play for infants who could not sit or stand by themselves involved 20 min of playing with toys in sitting and standing positions with support around the pelvic area and a 10 min seated session of listening to a storybook, singing a song, clapping hands, and enjoying peek-a-boo. The 30 min play for infants who could independently sit and stand was 20 min of active playing with the toys in sitting, standing, and cruising modes and a 10 min session of walking with support outside the building once a week and a 10 min session of listening to a storybook, singing a song, clapping hands, and enjoying peek-a-boo twice a week. The 15 min free moving on the floor allowed infants to move in and out of various positions such as creeping, crawling, sitting, pulling to stand, or standing with supervision. The stimulation program was performed in a group of 2–4 persons each time by pediatric physiotherapists who have more than 5 years of experience working with orphaned infants. In the upright position, each therapist supported each infant who could not sit or stand independently. Toys were used for movement motivation in these sessions. Being in an upright position was performed when infants were well and in cooperation, such as infants could sit while supported by holding their head in midline or standing with support bearing their weight on their feet or with variable movement of legs [35]. The play activities in an upright position were stopped if the infant cried or showed any unhappiness.

#### 2.5.2. Routine Care for Both Groups of Participants

All infants received routine care from experienced caregivers at each orphanage. The routine care was composed of a 5 min massage session and free play on the floor in supine, prone, or seated postures for approximately 90 min/time, 1 or 2 times a day. Each infant received regular bathing once or twice a day, diaper changing when needed, and feeding at lunch and dinner time.

### 2.6. Procedure

The collection of data for the study was conducted at the orphanages from December 2019 to September 2021. Characteristic data of participants, including gender, birth weight, birth height, head circumference, gestational age, and Apgar score at 5 min, were recorded from infants’ history files. The main caregivers responsible for childcare of children aged newborn to 12 months at the orphanages were interviewed about their level of education, child-raising experience in the orphanage, and age. The infant-to-caregiver ratios during the data collection period were also recorded.

Gross motor development and language skills were tested on 4 time points, such as baseline measures prior to the 12-week intervention and after 4-, 8-, and 12-week interventions. The first data collection for each infant was performed 15 days after recruitment. The observer got familiar with infants. Infants’ gross motor development was observed and recorded according to each posture using the AIMS Thai version [37] while an infant was moving in their familiar environment and had their caregivers nearby. The observation time was approximately 20 min for each infant. The main caregiver of each orphanage who is familiar with all infants and routinely raised infants was asked to go through the checklist of infants’ early communication and observe infants’ symbolic behaviors using the CSBS-DP. This took approximately 10 min. Only a single caregiver observed the communication skills and filled out the checklist for all infants. Scores of gross motor and communication behaviors were blindly summed and analyzed.

As mentioned earlier in the introduction, the intra-individual variability of gross motor, fine motor, and language developments or changes in percentiles of developments from four time points were calculated as the maximum percentile value minus the minimal percentile value [15].

### 2.7. Data Analyses

The characteristics of participants, developmental raw scores, developmental percentiles, and data of main caregivers were described using descriptive statistics. Data analyses were performed with SPSS version 17.0 for Windows (licensed by Khon Kaen University, Khon Kaen, Thailand) with a significance level of *p* < 0.05. The normal distribution of data was analyzed using a Shapiro–Wilk test. The range (maximum value–minimal value) of percentiles of development was analyzed to investigate the intra-individual variability. The gross motor development data for both groups in the current study were found to be normally distributed, and an independent *t*-test was therefore used to analyze the differences in the range of percentiles for gross motor development between the two groups. Because the language development data of both groups in the current study were not normally distributed, the Mann–Whitney U test was used to analyze differences in the range of percentiles for language development between the two groups. The levels of effect size convention were analyzed and classified as <0.49 = small, 0.50–0.79 = medium, and ≥0.80 = large [39]. Correlations of intra-individual variability between gross motor and language developments were analyzed using Spearman’s rank correlation (r_s_). The level of relationship was classified according to Portney et al. (2009) as follows: <0.25 = no correlation, 0.25–0.50 = fair, 0.50–0.75 = moderate to good, and >0.75 was good to excellent correlation [40].

## 3. Results

The main results found that there were significant differences in intra-individual variability of gross motor and language developments between the experimental and the control groups (Table 3). The experimental group exhibited significantly wider intra-individual variability of gross motor and language developments compared to the controls with medium to large effect size (d_cohen_ = 0.74–0.84). The intra-individual variability of language development in the control group was significantly narrower; however, the result shows that the standard deviation was wider, indicating the greater distribution of data in the control group.

Table 4 shows the mean (SD) percentiles of development at each time point of assessment: gross motor and each composite of language in the experimental and control groups. Considering data cross-sectionally, the gross motor percentile of infants in the experimental group appears to increase after each month of intervention, from 36.6% to 64.6%, which is greater than the 50th percentile after each month following the stimulation program, while infants in the control groups showed an increase in gross motor percentile from 42.5% to 59.4%. There were no cases in which the mean value of the gross motor development percentile was below the risk point (less than 10%) of gross motor developmental delays at any time point.

The overall mean percentile of language development of infants in the experimental group ranged from 25.5% to 36.4%. Although the mean language percentiles of infants in the control group were slightly increased, the mean value was less than the 16th percentile at all time points. Mean values of speech composite showed slight instability. However, the symbolic composite function of language development appears lower than the 16th percentile most of the time in both groups. There was a significantly moderate correlation of intra-individual variability between gross motor and language developmental percentiles of infants in the experimental group (r_s_ = 0.541 (*p* = 0.030)), while there was no correlation of intra-individual variability between gross motor and language developmental percentiles of infants in the control group (Table 5).

## 4. Discussion

The aim of this study was to investigate the effect of structured play in upright positions on intra-individual variability of the gross motor and language skills in institutionalized infants and examine the correlation of intra-individual variability between the gross motor and language skills during the age of 6–10 months. The main results showed significant differences in intra-individual variability of gross and language developments over the 3-month period between the experimental and control groups. The 45 min structured program was designed with an emphasis on infants interacting with objects and their environment with the opportunity to be in upright postures such as through sitting, standing, or cruising. Infants could freely move around or interact with caregivers or surroundings. Infants in the experimental group had a significantly wider range of intra-individual variability of gross motor development, which could be an effect of the additional play activity and the opportunity provided to control their trunks in upright positions. Recruited infants aged 6 to 7 months in this study were not able to independently sit or stand when they first joined the upright play program. An appropriate level of support for infants to be in sitting or standing postures within their familiar circumstances helped infants learn to control their bodies and balance themselves in vertical postures. A previous observational study of healthy full-term infants investigated the attainment of sitting at 5 months of age across different cultures of home rearing, providing new insights into varied opportunities for practicing motor ability and the social contexts in sitting ability. Researchers suggested that providing varied opportunities for practice and contextual factors of child-rearing influence the proficiency of infant sitting skills [36]. Accordingly, the modification of daily activity and the environment for practicing movement in healthy infants in orphanages helps with acquiring new age-appropriate skills.

After participating in the play program for a 3-month period, healthy orphaned infants in this study were able to show wider intra-individual variability of gross motor developmental percentiles. The wide intra-individual variability was in line with previous longitudinal studies indicating that developing infants typically exhibit variability in terms of gross motor percentile from 3 to 13 months [11,12,13,33,41], while intra-individual variability of gross motor development in the control group was quite stable, similar to previous studies on orphaned infants who displayed narrow intra-individual variability [19,20]. The mean value of intra-individual variability within 12 months in the study of Prommin et al. was 55.4 percentile, while the mean intra-individual variability within 3 months of the current study was 34.3 percentile (Table 3). Although the participants in the control group had no biological risks, their intra-individual variability was still narrower compared with the experimental group. It can be presumed that infants raised in a limited environment have less opportunity to experience various movements. This supports the suggestion by Adolph et al. (2010) that restricting practice or reducing stimulation at appropriate time points within the circumstances of child-rearing practice, such as being raised in orphanages, can delay developmental milestones [42].

According to a number of studies [42,43], infants aged from 6 months and up to the end of their first year are able to attain new skills related to being upright, such as crawling, sitting up, pulling to stand, cruising, and finally walking independently. At this age range, a child is able to independently control the lower body and pelvis in conjunction with the development of past upper trunk or upper chest motor skills in the prone position [35]. The postural transition around 9–10 months encourages preparation for the development of standing and walking [44]. In contrast, orphaned infants are raised in limited spaces within their cribs, deprived of sensory stimulation due to being raised in a closed area, fed with a bottle propped on a pillow, and barely handled by caregivers or only interacted with them for brief diaper changes. Such child-rearing practices in these environments can lead to marked variability in terms of physical, social emotions, and global development, which could affect language development.

In addition, this current study found a significantly larger range of intra-individual variability of language development in the experimental group. During the upright play activity, participants in the experimental group were motivated by colorful toys and interaction with two-way communication. The activities with two-way communication, such as singing and clapping hands, peek-a-boo, and story-listening activities, were performed by a pediatric physical therapist who is familiar with infants. The activity is different from putting infants in a child seat and listening to music normally provided at children’s homes. Typically, infants at the age of 6 to 8 months show early skills of communication by producing polysyllabic babbling, with the same syllable repeated over and over. By 9 months, infants sporadically produce truncated utterances, such as mama or dada, without comprehension of the meaning that adults have attached to these sounds. By 10 months of age, infants produce these utterances spontaneously and understand the meaning of words [45]. Therefore, around 6 to 12 months of age is an appropriate time for infants to be stimulated with early communication skills or have chances to interact with their caregivers, which helps to build and strengthen the developed bonding attachment between them. Through interaction with caregivers or parents, infants can learn to understand facial movements. Gallagher suggested that neonates’ spontaneous facial movements are arranged with their understanding of facial movements from the time they are born, resulting in awareness of facial expressions, and this can facilitate language development [46]. Furthermore, it is suggested that the parent-infant relationship has the greatest influence on infant development [47].

The results of language development at each time point (Table 4) at less than 16% may indicate delayed language skills in infants from the control group. Moreover, the social composite function of the experimental group at baseline measurement displays a higher mean value of development percentile compared with the control group. This could be explained by the general observation that child-rearing practices in the experimental group were different from those of the control group. The caregivers in the experimental group interacted or talked to infants during daily activity more than caregivers in the control group. From this point of view, it promotes the necessity for longitudinal assessments to evaluate child development. Language development is complex and requires more time to develop in young infants of this age. Longitudinal assessments or measuring variability within individuals could therefore provide a better picture of how infants learn to develop these skills. Interestingly, the mean percentiles of speech and the symbolic composite function of language skills did not differ between groups, and the symbolic composite function showed values below the cutoff point in both groups, which could be due to different factors such as infants may need a longer period than 3 months for intervention and that their symbolic skill is not yet developed at this age range. A previous study found that symbolic functions such as understanding and object use are developed at older than 18 months of age [38]. Future studies should consider providing more specific language skills of symbolic task training, for example, asking infants where is your tummy or give me the ball, to put your hands up, stack many blocks, or participate in role-playing with toys to stimulate understanding and object use.

Our longitudinal study revealed a significant moderate correlation of intra-individual variability between gross motor and language developmental percentiles during 6–10 months in the experimental group. A positive relationship between the intra-variability of gross motor and language implies that both skills can be varied when the environment is adjusted. Muluk et al. [48], in 2016, also found that language items were mostly correlated at 6 and 12 months. Both speech sounds and producing syllables were associated with social and gross motor development at 6 months. Furthermore, at 12 months, gross motor items such as standing by holding on were positively correlated with producing syllables for more than four words in the language items [48]. However, no correlation was found between the two variables in the control group of this study, displaying that the language development percentile is stable below the cutoff point (<16 percentiles). Environmental factors have a great potential to influence the development of language skills [18]. Infants who have an older sibling with autism spectrum disorder (ASD) and at-risk factors for motor and communication delays display stability of low scores of developments [30]. Our study was in contrast to the results of a previous study that found the relationship between the intra-variability of motor and language percentiles to be nonsignificant [12]. A reason for this discrepancy could be the differences in the age ranges of participants. The Darrah et al. 2003 [12] study investigated the communication in home-raised infants at the age of 13 months upwards, while infants in this study were those residing in the orphanage during 6–10 months of age. However, infants with at-risk factors for motor and communication delays should be supported in developmental surveillance or the process of serial intervention in a child’s development over time. From our results, it is possible that gross motor and language development may show their relationship at a certain age range, but the relationship can be unstable depending on the variation of each developmental domain and environment. We suggest that each domain of development should be simultaneously promoted during child-rearing practices. According to the commentary theory of motor development [14], the modified environment can influence the functional motor solution infants use to accomplish a motor skill. Interaction between child and caregiver could help to improve communication skills. Therefore, functional gross motor and communication solutions are influenced by environmental constraints and facilitators during child-rearing.

Although significant results were found in this study, there are nonetheless several limitations. The number of recruited participants did not meet the required sample size calculation. The post hoc power of testing 16 infants in the experimental group and 14 infants in the control group was 0.50 in gross motor variability development. For language development, the post hoc power of testing was 0.60. Moreover, the participants were recruited from three orphanages in Thailand, and further research on a larger sample size of orphaned infants living in a similar or the same physical environment and characteristics of caregivers is needed. Therefore, broad conclusions should be drawn cautiously. The upright-play program is suitably designed for healthy orphaned infants, and an investigation of the effects of a similar program on intra-individual variability of infants who have biological risks is required. Furthermore, the stimulation program should be performed by the main caregivers of the orphanages in future studies. Alternatively, the program should be taught to experienced caregivers working in the orphanage. Finally, but importantly, the language skills should be followed for a longer period of time because more time is needed for this skill to develop, especially in infants aged less than 2 years.

## 5. Conclusions

In the present study, an additional stimulation program of upright play was provided for infants aged 6 months onwards that were living in orphanages. The additional program of the 45 min play in an upright position was combined with activities of social interaction between infants and caregivers and exploring the environment outside the crib. Within a period of 3 months, infants showed significantly wider ranges of intra-individual variability of gross motor and language performances compared with those who did not receive the stimulation program. We suggest that modification of the environment during child-rearing of orphaned infants for 3 months could lead to dynamic changes in the individual variability of developmental skills, as measured by longitudinal assessments. The study also found a significant correlation of intra-individual variability between gross motor and language development in infants aged 6 to 10 months old when child-rearing practices included environmental modifications. This study suggests that the correlation between gross and language development could be changed according to the age range and child-rearing practice. Therefore, in order to boost these skills in infants living in orphanages, both gross motor and language development should be simultaneously promoted.

## Figures and Tables

**Table 1:** Infant characteristics.

Characteristics	Experimental Group (n = 16)	Control Group (n = 14)
Gender (n, boys/girls)	10/6	6/8
Age at recruitment - 6 months - 7 months	8 8	9 5
Birth weight (g)	2782.3 (319.8)	2789.0 (192.7)
Birth height (cm)	49.1 (2.5)	49.4 (1.9) ^#^
Birth head circumference (cm)	31.6 (1.5)	31.9 (1.2) ^#^
Gestational age (weeks)	37.9 (0.8)	37.9 (0.9) ^#^
Apgar score at 5 min	9 (0.7) ^$^	9 (0.8) ^#^
Age at admission in the orphanage (days)	50 (28)	90 (40)

^$^ No information on Apgar score at 5 min for 1 infant in the experimental group. ^#^ No information on birth height, birth head circumference, gestational age, and Apgar score for 4 infants in the control group.

**Table 2:** Characteristics of main caregivers in orphanages.

Characteristics	Experimental Group (n = 8)	Control Group (n = 7)
Caregiver’s education, n (%)		
• Lower than bachelor’s degree	6 (75.0%)	4 (57.2%)
• Bachelor’s degree	2 (25.0%)	3 (42.8%)
Child-raising experience in orphanages (years), mean (SD)	17.9 (11.7)	16.5 (8.0)
Age (years), mean (SD)	46.9 (13.6)	43.7 (11.5)
Caregiver to child ratio	1:8–12	1:7–10

**Table 3:** Mean (SD) of intra-individual variability (changes) of developments.

Developments	Experimental Group (n = 16)	Control Group (n = 14)	*p*-Value	Effect Size (d_cohen)_
Gross motor	45.4 (16.3)	34.3 (13.6)	0.042 *	0.74
Language	33.9 (16.6)	17.1 (23.0)	0.009 **	0.84

* *p*-value < 0.05 (independent *t*-test) and ** *p*-value < 0.01 (Mann–Whitney U test).

**Table 4:** Mean (SD) of developmental percentiles at each time point.

Developments	Experimental Group (n = 16)			
	Pre-Test	Post 4-Week Intervention	Post 8-Week Intervention	Post 12-Week Intervention
Gross motor	36.6 (23.1)	54.4 (24.0)	67.8 (21.1)	64.6 (22.6)
Language	25.5 (22.4)	29.4 (33.6)	28.5 (21.0)	36.4 (23.3)
- Social	53.7 (31.8)	49.2 (32.4)	54.7 (23.8)	58.7 (30.1)
- Speech	28.8 (23.8)	32.7 (27.4)	35.9 (22.7)	25.3 (27.1)
- Symbolic	3.3 (1.8)	11.4 (16.9)	15.2 (4.5)	17.0 (13.4)
**Developments**	**Control group (n = 14)**			
Gross motor	42.5 (30.1)	52.4 (22.6)	59.4 (22.2)	45.5 (24.0)
Language	7.2 (8.5)	8.8 (17.2)	11.6 (19.0)	13.8 (23.5)
- Social	28.8 (23.8)	32.7 (27.4)	35.9 (22.7)	25.3 (27.1)
- Speech	23.3 (22.2)	24.6 (26.7)	22.9 (20.5)	21.6 (10.5)
- Symbolic	2.7 (1.8)	8.5 (10.9)	13.4 (25.4)	11.7 (17.5)

**Table 5:** Correlation of intra-individual variability between gross motor and language developments.

Developments	Experimental Group (n = 16)	Control Group (n = 14)
	Gross Motor	Gross Motor
Language	0.541 * (*p* = 0.030)	−0.061 (*p* = 0.837)

* *p*-value < 0.05.
